# Relationship of Self-Rated Health with Fatal and Non-Fatal Outcomes in Cardiovascular Disease: A Systematic Review and Meta-Analysis

**DOI:** 10.1371/journal.pone.0103509

**Published:** 2014-07-30

**Authors:** Nahal Mavaddat, Richard A. Parker, Simon Sanderson, Jonathan Mant, Ann Louise Kinmonth

**Affiliations:** 1 Primary Care Unit, Department of Public Health and Primary Care, University of Cambridge, Strangeways Laboratory, Cambridge, United Kingdom; 2 Primary Care Unit, Department of Public Health and Primary Care, Institute of Public Health, University of Cambridge, Cambridge, United Kingdom; Innsbruck Medical University, Austria

## Abstract

**Background:**

People who rate their health as poor experience higher all-cause mortality. Study of disease-specific association with self-rated health might increase understanding of why this association exists.

**Objectives:**

To estimate the strength of association between self-rated health and fatal and non-fatal cardiovascular disease.

**Methods:**

A comprehensive search of PubMed MEDLINE, EMBASE, CINAHL, BIOSIS, PsycINFO, DARE, Cochrane Library, and Web of Science was undertaken during June 2013. Two reviewers independently searched databases and selected studies. Inclusion criteria were prospective cohort studies or cohort analyses of randomised trials with baseline measurement of self-rated health with fatal or non-fatal cardiovascular outcomes. 20 studies were pooled quantitatively in different meta-analyses. Study quality was assessed using Newcastle-Ottawa scales.

**Results:**

‘Poor’ relative to ‘excellent’ self-rated health (defined by most extreme categories in each study, most often’ poor’ or ‘very poor’ and ‘excellent’ or ‘good’) was associated over a follow-up of 2.3–23 years with cardiovascular mortality in studies: where varying degrees of adjustments had been made for cardiovascular disease risk (HR 1.79 (95% CI 1.50 to 2.14); 15 studies, I2 = 71.24%), and in studies reporting outcomes in people with pre-existing cardiovascular disease or ischaemic heart disease symptoms (HR 2.42 (95% CI 1.32 to 4.44); 3 studies; I2 = 71.83%). ‘Poor’ relative to ‘excellent’ self rated health was also associated with the combined outcome of fatal and non-fatal cardiovascular events (HR 1.90 (95% CI 1.26 to 2.87); 5 studies; I^2^ = 68.61%), Self-rated health was not significantly associated with non-fatal cardiovascular disease outcomes (HR 1.66 (95% CI 0.96 to 2.87); 5 studies; I2 = 83.60%).

**Conclusions:**

Poor self rated health is associated with cardiovascular mortality in populations with and without prior cardiovascular disease. Those with current poor self-rated health may warrant additional input from health services to identify and address reasons for their low subjective health.

## Introduction

Self-rated health (SRH), a simple measure of subjective health status, strongly and consistently predicts all-cause mortality across varied populations even after adjustment for demographic, biophysical, and behavioural risk factors. [1], [2] The reasons behind this are uncertain. SRH may be a sensitive reflection of current underlying disease or proxy for a subclinical state - a measure of the awareness of symptoms, disease or risk factors; or alternatively a reflection of personal characteristics that may impact upon future health outcomes [2]–[5].

Studies of SRH have primarily studied its’ relationship with all-cause mortality.[1] All-cause mortality is objective, relatively easy to collect and comparable across populations. It is, however, a blunt instrument for analysing causal pathways. The study of SRH in specific diseases may be an important step in the understanding of the relationship between SRH and mortality. A further benefit of shifting the focus of SRH research to its relationship with specific disease incidence and disease outcomes is that better specification and measurement of relevant covariates may also provide more precise estimates of any independent effect of SRH. Such information could be used to assess the potential use of SRH for risk prediction in specific diseases. In addition, important differences may exist in the relationship between self-rated health and mortality among those with and without specific pre-existing disease, which may be obscured in available studies which have not characterized populations in this way, focusing mainly on large, heterogeneous samples of national or regional populations. [6] For example, Idler et al found that SRH predicts subsequent mortality more strongly in those with circulatory system disease than in those with no identified cardiovascular condition. [7].

The relationship between SRH and mortality has been shown to vary by disease. In a unique analysis of 700,000 American National Health Interview Survey participants followed for over 20 years, self-rated health strongly predicted death from diabetes, infectious, and respiratory diseases, (HR of 6.1, 3.7, 3.7 respectively), and a to a lesser extent from coronary heart disease (HR of 2.3) and cancer (HR of 1.6) [6] A more recent study in 2013 of 4770 mid-life adults participating in the US Health and Retirement study also found SRH to be a significant predictor of onset of chronic conditions including coronary heart disease, stroke, diabetes, lung disease and arthritis. [8] This link between SRH and morbidity is, however, less certain and it is unclear whether the relationship between SRH and all-cause mortality is mediated through disease-specific morbidity pathways.

This meta-analysis was undertaken to collate evidence from individual studies and examine the association across studies between SRH and cardiovascular disease-specific mortality and morbidity. Cardiovascular disease including stroke was chosen since it remains the most important cause of morbidity and mortality in many countries and because its epidemiology is well established, facilitating better measurement and control of covariates in study design and analysis. It has been speculated that the association of poor self-rated health with all-cause mortality may be driven by its association with cardiovascular diseases.[9] No formal meta-analyses have previously explored the relationship between SRH and CVD mortality or morbidity.

## Materials and Methods

A comprehensive search of the following electronic resources was undertaken during June 2013: PubMed MEDLINE, EMBASE, CINAHL, BIOSIS, PsycINFO, DARE, Cochrane Library, and Web of Science. Search strategies were tailored to each database using a combination of indexed headings and text words; a sample search is included in the Appendix. Two reviewers independently searched the databases, selected the studies and reviewed the contents of the manuscripts to determine whether they met the criteria for inclusion. Data were then independently extracted and quality assessment performed. When discrepancies occurred between reviewers in determining inclusion into the analyses, other authors were asked to evaluate the studies. No language or time restrictions were imposed. Authors of primary studies and experts in the field were contacted to answer questions about methodology or study results. For the systematic review, studies had to meet the following inclusion criteria: (a) adult populations (b) current self-rated health measured by a single question at the beginning of follow-up, with clearly defined and similar response categories (c) prospective cohort study, cohort analysis of randomised trials or incident (nested) case-control study (d) reporting non-fatal events and/or fatal outcomes for coronary heart disease, stroke, or combined CVD. Assessment of study quality was primarily concerned with identifying risk of bias, rather than merely examining the quality of article reporting [10] using the Newcastle-Ottawa scales. [11].

Studies were sub-divided into those reporting: (i) fatal CVD events or mortality outcomes in both population studies and in those reporting outcomes in people with pre-existing cardiovascular disease or ischaemic heart disease symptoms (ii) those with non-fatal CVD events only (iii) and those with combined fatal and non-fatal CVD events.

We combined log-hazard ratios for appropriate subsets of individual studies using random-effects meta-analyses, and presented corresponding forest plots of hazard ratios with a diamond indicating the summary effect estimate and 95% confidence interval. Box sizes were drawn inversely proportional to the magnitude of the standard error for each study and indicate the relative weight of each study in the meta-analysis. Where there was more than one CVD outcome per study or analyses on two or more patient samples, the effect sizes for each outcome/patient sample were averaged using a fixed effects meta-analysis to generate a single effect size for each study. The meta-analysis focussed on the comparison of patients reporting poor health relative to excellent health, via hazard ratios. For studies analysing ordered categories, we used the categories at each end of the Likert scale (labelled as “excellent” self-rated health - reference category- and “poor” respectively). A number of studies collapsed their self-rated health categories into a dichotomous variable, likely to ensure adequate numbers for analysis. For these, the highest SRH category analysed was used as the “excellent” reference category and the comparison category labelled “poor” self-rated health. Heterogeneity between studies was assessed using the Q-test for heterogeneity and I^2^-statistic, based on the DerSimonian-Laird estimator. Publication bias was assessed using a funnel plot and a rank correlation test for funnel plot asymmetry. [12] Sensitivity analyses were performed excluding studies that had used different patient populations or non-standard outcome measures. The ‘metafor’ package in R software (R Development Core Team, 2012) was used to conduct all meta-analyses.[13], [14] The article was drafted using the PRISMA reporting guidelines. [15].

## Results

**Figure 1:**
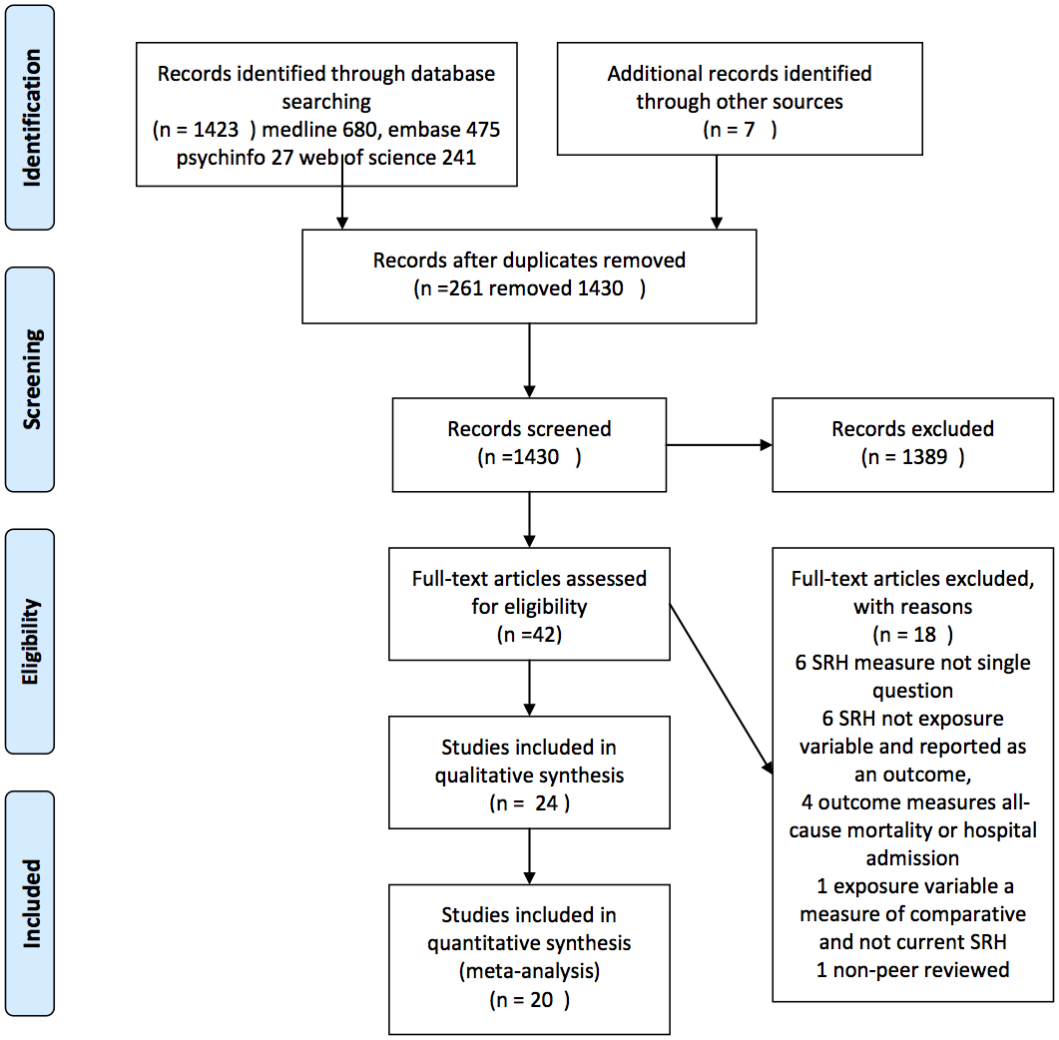
Prisma Flow Diagram.

Figure 1 shows the number of records identified, screened and excluded. 42 studies were identified as being potentially relevant (including four non-English articles which were translated), of which 24 met the inclusion criteria. Four of these were subsequently excluded from the meta-analyses: two were matched case-control studies with results expressed as conditional odds ratios; [16], [17] one only reported proportional hazard ratios and inexact p values; [8] and one only reported on combined risk of stroke recurrence and death in a stroke population. [18] Appels (1996) reported two sets of results for Lithuania and the Netherlands. These were treated as separate studies because the analysis was applied to two distinct populations. [9].

### Study characteristics and participants

**Figure 2:**
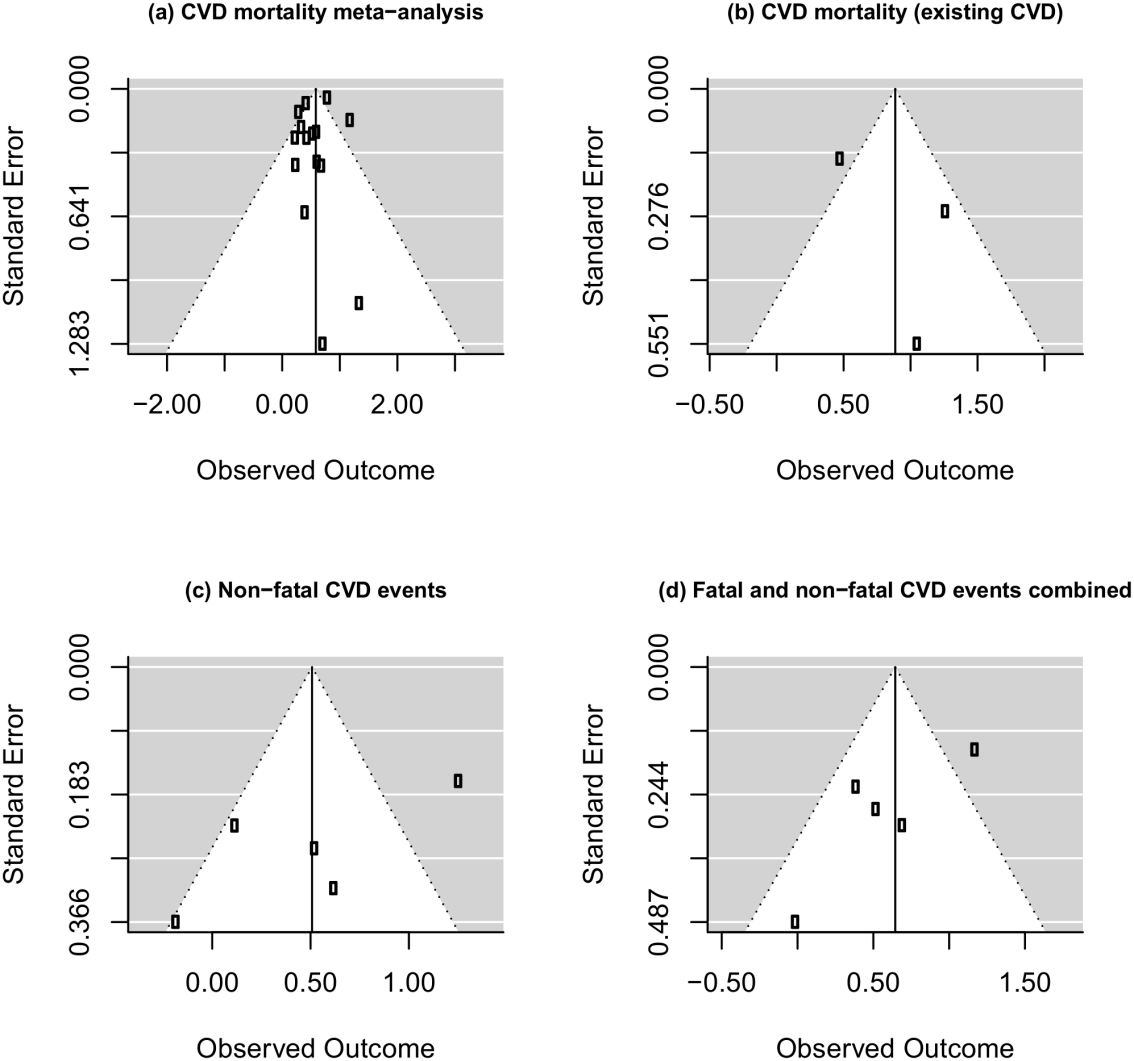
Funnel plot of standard error against effect size (log hazard ratio) for (a) CVD mortality meta-analysis [Kendal’s tau = −.20, p = .82] (b) CVD mortality meta-analysis (existing CVD) [Kendal’s tau = −.33, p = 1.00] (c) Non-fatal CVD events meta-analysis [Kendal’s tau = −.24, p = .24] (d) Fatal and non-fatal CVD events combined meta-analysis: [Kendal’s tau = −.24, p = .24].

Details of the 20 studies included in the various meta-analyses are presented in Table 1. [6], [7], [9], [19]–[34] Thirteen populations studied were in Europe, 5 in the USA and 2 in Asia. Sample sizes ranged from 234 to 689,710 and follow-up ranged from 2.3–23 years, with most studies reporting results from more than 5 years follow-up. Study participants were mostly middle-aged or older adults, with six studies conducted exclusively in men, [9], [26], [27], [30], [34] and two exclusively in women.[29], [31] Other than three studies based on persons with pre-existing cardiovascular disease or ischaemic heart disease symptoms, [7], [19], [31] the focus was on populations without, or controlling for previous CVD events or risk. Characterisation of populations varied with the majority assessing sociodemographic factors and classic CVD risk factors (blood pressure, blood glucose, lipids, alcohol, BMI, smoking, physical activity) but varying in inclusion of other factors such as education or socioeconomic status [7], [20], [33] and diagnoses of other diseases such as depression [19], [21], [28], [29], [31] or diabetes [9], [19], [21], [29], [32]. Only two studies included family history of CVD. [30], [33] One study assessed objective measures of lifestyle with vitamin C levels. [33] Health service utilisation was not reported in any of the studies. CVD mortality was the predominant outcome reported; five studies separately reported incidence of a non-fatal CVD event.[9], [20],[28],[33] There was generally poor measurement of disease severity in studies. Table 2 outlines the risk of bias in included studies based on the Newcastle-Ottawa Quality Assessment Scales. Visual inspection of the funnel plots for each analysis (figure 2) and the results of the rank correlation tests suggest that publication bias was unlikely, with an even distribution of study effect sizes plotted against precision in the funnel plots and relatively high *P*-values in the statistical tests. Sensitivity analyses are shown in Table 3.

**Table 1:** Included study characteristics.

Study & yearpublished§	Country	Study Sample	Years followUp	SRH questiontype (numberof responseoptions) £	PopulationBaseline CVDstatus	Baseline CVDrisk factors &measurement	Cardiovascular Outcomesmeasured	Analysisadjustments
Appels(Kaunas)1996(11)	Lithuania	n = 2,452 100%males Aged45–60	10 (Approx)	Current (5) Agecomparative (3)	Not reported	Age, BMI,cholesterol, BP,glucose tolerance,smoking, alcohol,physical activity	CVDMortalityIncidencenon-fatalAMI	Baseline CHD andCVD risk factors
Appels(Rotterdam)1996(11)	Netherlands	n = 3,365 100%males Aged45–60	10 (Approx)	Current (5) Agecomparative (3)	Not reported	Age, BMI,cholesterol, BP,glucose tolerance,smoking, alcohol,physical activity	CVD MortalityIncidencenon-fatalAMI	Baseline CHD andCVD risk factors
Benjamins2004(6)	USA	n = 689,71046.2% malesMean age 44.4	7 (Mean)	Current (5)	Not reported	Demographics,BMI	CHD mortalityStroke mortality	Demographics andBMI
Bosworth$1999(23)	USA	n = 2,885 68%males Meanage 62.5	3.5 (Mean)	Current (5)	ObjectivelydiagnosedCHD	Diabetes,hypertension,stroke, depression,smoking, severity,demographics	CHD mortality	Multi-morbidity,severity andpsycho-social factors
Doğanay2012 (24)	Turkey	n = 1,382 39.2% males Aged 65–74	2 years	Current (5)	Free of CVD	Framingham RiskSocioeconomicstatus	Coronaryevents andCHD mortality	Framingham riskand socioeconomicstatus
Ernsten 2011(25)	Norway	n = 5,808 55.7%males Over70 years	7.9 (Mean)	Current (4)	Free of CVD	Social networks,physical activity,smoking,depression, anxiety,BP, diabetes	CHD mortality	CVD risk factorsand depression
Fang2003(26)	China	n = 3,15748.7% males	8 (Total)	Current (3)	Reported	Demographics,BMI, BP	CHD mortalityStrok mortality	Baseline CVD statusbut not risk factors
Fernandez-Ruiz 2012(27)	Spain	n = 4,958 42.5%males	13 (Total)	Current (5) Agecomparative (5)	Recorded	Demographic,lifestyle, physicalactivity, alcohol,smoking	CVD mortality	Sociodemographic,functional statusand comorbidities
Heidrich*2002(28)	Germany	n = 3,019 50.4%males Meanage 47.1	10.9 (Median)	Current (4) Agecomparative (4)	Reported	Smoking, alcohol,physical activity,demographics, BP,lipids, BMI	CVD mortality	Baseline CVD andrisk factors
Heistaro2001(29)	Finland	n = 21,30248.6% malesMean age 42.9	23 (Total)	Current (5)	Reported	Smoking, physicalactivity, age, BMI,lipids, BP	CVD mortality	Baseline CVD andrisk factors
Idler$2004(7)	USA	n = 3,709 45.8%males Meanage 48.3	20 (Total)	Current (5)	Sample 2:Self-reportedCVD	CVD anddemographics,education, income	CVD mortality	Stratified analysis
Kamphuis2008(30)	Finland,Italy,Netherlands	n = 909 100%males Meanage 76.4	10 (Mean)	Current (5)	Reported	Smoking, physicalactivity, BMI,lipids, BP	CVD mortality	CVD or diabetesexcluded CVD riskfactors, physicalactivity anddisability
Kaplan1996(31)	Finland	n = 2,512 100%males Aged 42,48, 54, 60 atrecruitment	5.8 (Mean)	Current (5)	Reported	Smoking, physicalactivity,demographics,BMI, lipids, BP	CVD mortalityIncidence fataland non-fatalAMI combined	Symptomatic andasymptomaticdisease CVD riskfactors
Kennedy2001(32)	USA	n = 2,810 41.6%males Over 65at recruitment	15 (Total)	Current (5)	Reported	Smoking, alcohol,BMI, depression,demographics,prior diseases	Non-fatalhospitalisationfrom: AMIHeart failure	Self-reporteddiseaseand CVDrisk factors
Kuper2006(33)	Sweden	n = 48,066 100%females Meanage 40.3	11.3 (Mean)	Current (4)	Not reported	Age, smoking,alcohol, BMI,depression,physical activity,diabetes	Incidence fatalCHD ornon-fatal AMIcombined	CVD risk factors
Pijls 1993(9)	Netherlands	n = 783 100%males Aged65–85 atrecruitment	5 (Total)	Current (4)	Reported	Smoking, physicalactivity, alcohol,demographics,BMI, lipids, BP,family history	CVD 5 yearmortalityIncident firstfatal andnon-fatal CVDevent combined	Baseline CVD andrisk factors
Rutledge2010 (34)	USA	n = 936 100%femalesOver 18	5.9 (Median)	Current (5)	Symptomaticwomenreferred forangiography	Physical activity,education,smoking,depression, anxiety,lipids, BMI, BP,diabetes	Fatal andnon-fatalCVD eventscombined	Age, CVD riskfactors, symptoms,knowledge ofangiogram results
Tsuji1994(35)	Japan	n = 2,552 44.6%males Aged65–113 atrecruitment	2.9 (Mean)	Current (4)	Reported	Demographics,diabetes,hypertension	CHD mortalityStroke ortality	Baseline CHD andstroke excluded Useof medical care
van der Linde2013 (36)	UK	n = 20,94143.4% malesAged 39–74	11 (Mean)	Current (4)	Reported	Sociodemographic,cholesterol,BP, BMI, familyhistory, smoking,alcohol, physicalactivity, vit C	Fatal andnon-fatal CVDevents	Sociodemographic,behavioural andclinical
Wannamethee1991(37)	UK	n = 7,275 100%males Aged40–59 atrecruitment	4 (Mean)	Current (4)	Not reported	Smoking, physicalactivity, alcohol,BMI, lipids, BP	CVD mortality	With or withoutrecall of any majordiagnosis Age

**Key:** SRH = self-rated health; CVD = cardiovascular; CHD = coronary heart disease; MI = myocardial infarction; BMI = body mass index; AMI = acute myocardial infarction; BMI = body mass index; BP = blood pressure; SBP = systolic blood pressure; HDL = high density lipoprotein; LDL = low density lipoprotein; ADL = activities of daily living; MMSE = mini-mental state examination.

*Heidrich only reported results of CVD deaths for males.

£SRH Current – current health status Age comparative – compared to those of own age.

$Bosworth recruited only patients with prevalent CHD or stroke respectively; Idler results reported for self-reported circulatory disorders and newly diagnosed circulatory disorders.

∧Percentages not directly comparable as some studies did not report separate data for the worst category of SRH.

**Table 2:** Quality Assessment of included studies based on the Newcastle-Ottawa Scales.

Study	Howrepresentativewas the exposedcohort?	Selection ofnon-exposedcohort	Ascertainmentof exposure	Demonstrationthat outcome ofinterest was notpresent at startof study	Of cohorts onbasis of designor analysis		Assessmentof outcome		Follow uplong enoughfor outcomesto occur	Adequacy ofcohort follow-up
Appels (11)	Somewhatrepresentativeof males aged45–60	Drawn from the same community as exposed cohort	From structured interview	No	Study controls for multiple covariates		Independent assessment from secure records		Yes	No description
Benjamins (6)	Representativenational sampleof adults	Drawn from the same community as exposed cohort	From structured interview	Yes	Study controls for multiple covariates		Independent assessment from secure records		Yes	Over 99% followed
Bosworth (23)	Somewhatrepresentativeof CHD patients	Drawn from the same community as exposed cohort	From structured interview	Yes	Study controls for multiple covariates		Independent assessment from secure records		Yes	No description
Doğanay (24)	SomewhatRepresentativeof adults aged65–74	Drawn from the same community as exposed cohort	Not available	Yes	Study controls for multiple covariates		Independent assessment from secure records		Yes	Not available
Ernsten (25)	Representativeof nationalsample adultsover 70	Drawn from the same community as exposed cohort	Written self-report	Yes	Study controls for multiple covariates		Independent assessment from secure records		Yes	No description
Fang (26)	Somewhatrepresentativesample of adultsaged 55 and over	Drawn from the same community as exposed cohort	Written self-report	Yes	Study controls for multiple covariates		Independent assessment from secure records		Yes	No description
Fernandez-Ruiz(27)	Representativesample of adultsaged 65 and over	Drawn from the same community as exposed cohort		Yes	Study controls for multiple covariates		Independent assessment from secure records		Yes	No description
Heidrich (28)	Somewhatrepresentativesample of adultsaged 25–64	Drawn from the same community as exposed cohort	From structured interview	Yes	Study controls for multiple covariates		Independent assessment from secure records		Yes	Complete follow-up
Heistaro (29)	Representativenational sampleof adults	Drawn from the same community as exposed cohort	Written self-report	Yes	Study controls for multiple covariates		Independent assessment from secure records		Yes	No description
Idler (7)	Representative ofpersons with self-reported CVD andnewly diagnosedCVD	Drawn from the same community as exposed cohort	From structured interview	Yes	Study controls for multiple covariates in different subgroups		Independent assessment from secure records		Yes	90% follow-up
Kamphuis (30)	Somewhatrepresentativesample of middle-aged men	Drawn from the same community as exposed cohort	Written self-report	Yes	Study controls for multiple covariates		Independent assessment from secure records		Yes	No description
Kaplan (31)	Somewhatrepresentativesample of elderlymen	Drawn from the same community as exposed cohort	Written self-report	Yes	Study controls for multiple covariates		Independent assessment from secure records		Yes	No description
Kennedy (32)	Representative sample of community elderly	Drawn from the same community as exposed cohort	From structured interview	Yes	Study controls for multiple covariates		Independent assessment from secure records		Yes	Small number lost due to missing data
Kuper (33)	Representativesample of womenaged 30–50	Drawn from the same community as exposed cohort	Written self-report	Yes	Study controls for multiple covariates		Independent assessment from secure records		Yes	Complete follow-up
Pijls (9)	Somewhatrepresentativesample of elderlymen	Drawn from the same community as exposed cohort	Written self-report	Yes	Study controls for multiple covariates		Independent assessment from secure records		Yes	Complete follow-up
Rutledge (34)	Representative of women with cardiac symptoms	Drawn from the same community as exposed cohort	Written self-report	Yes	Study controlled for CAD severity scores		Telephone self report and from secure records		Yes	Complete follow-up
Tsuji (35)	Somewhatrepresentative ofadults 65 and over	Same community as exposed cohort	Written self-report	Yes	Study controls for multiple covariates		Independent assessment secure records		Yes	No description
van der Linde (36)	Populationrepresentative ofadults 39–74	Drawn from the same community as exposed cohort		Yes	Study controls for multiple covariates		Independent assessment from secure records		Yes	No description
Wannamethee (37)	Representativenational sampleof men aged40–59	Drawn from the same community as the exposed cohort	Structured interview	Yes	Study controls for multiple covariates	Independent assessment from secure records		Yes		No description

**Table 3:** Sensitivity analysis – impact of excluding studies.

Studies included inmeta-analysis	No. ofstudies	Excluding	Summary estimate(95% CI)	I^2^-statistic
**Fatal CVD**				
All studies	15	None	1.79 (1.50 to 2.14)	71.24%
Well-controlled for confounders	13	Doğanay, Wannamethee	1.67 (1.41 to 1.98)	66.70%
Middle-aged or general population studies	8	Fernández-Ruiz, Doğanay, Ernsten, Kamphuis, Fang, Tsuji, Pijls	1.90 (1.51 to 2.39)	75.41%
Elderly population only (65 years and older expect Fang (2003) over 55 years)	7	van der Linde, Benjamins, Heidrich, Heistaro, Kaplan, Appels, Wannamethee	1.50 (1.27 to 1.76)	0%
Male populations only	9	van der Linde, Fernández-Ruiz, Doğanay, Benjamins, Fang, Tsuji	1.74 (1.36 to 2.23)	70.03%
Well-controlled studies based on male populations only	8	van der Linde, Fernández-Ruiz, Doğanay, Benjamins, Fang, Tsuji, Wannamethee	1.52 (1.34 to 1.72)	0%
Both male and female populations	8	Pijls, Appels, Kaplan, Heidrich, Kamphuis, Wannamethee	1.68 (1.32 to 2.12)	78.56%
All except Benjamins	14	Benjamins (non-proportional hazards)	1.73 (1.45 to 2.05)	51.26%
*Pre-existing Disease*				
All	3	None	2.42 (1.32 to 4.44).	71.83%
Excluding female studies	2	Rutledge	2.34 (1.09 to 5.06)	84.95%
				
**Non-fatal CVD**				
All studies	5	None	1.66 (0.96 to 2.87)	83.60%
Middle-aged patient population*	3	Kennedy, Doğanay	1.80 (0.81 to 4.02)	87.14%
Well-controlled for confounders	4	Doğanay	1.60 (0.82 to 3.14)	87.67%
All except Van der Linde (2013)	4	van der Linde (heavily influences between-study heterogeneity)	1.34 (0.97 to 1.86)	27.24%
All except Van der Linde (2013) and Doğanay (2012)	3	van der Linde, Doğanay	1.23 (0.85 to 1.77)	27.47%
				
**Fatal and non-fatal CVD**				
All studies	5	None	1.90 (1.26 to 2.87)	68.61%
Well-controlled for confounders	4	Doğanay	1.84 (1.10 to 3.09)	76.26%
Middle-aged patient population*	3	Pijls, Doğanay	2.08 (1.21 to 3.56)	79.42%
Male subjects only	2	Kuper, Doğanay, van der Linde	1.39 (0.92 to 2.08)	0%
All except Van der Linde (2013)	4	van der Linde	1.60 (1.21 to 2.13)	0%

*Using standard SRH scale only.

### Meta-analysis findings

#### 1. Fatal CVD events

**Figure 3:**
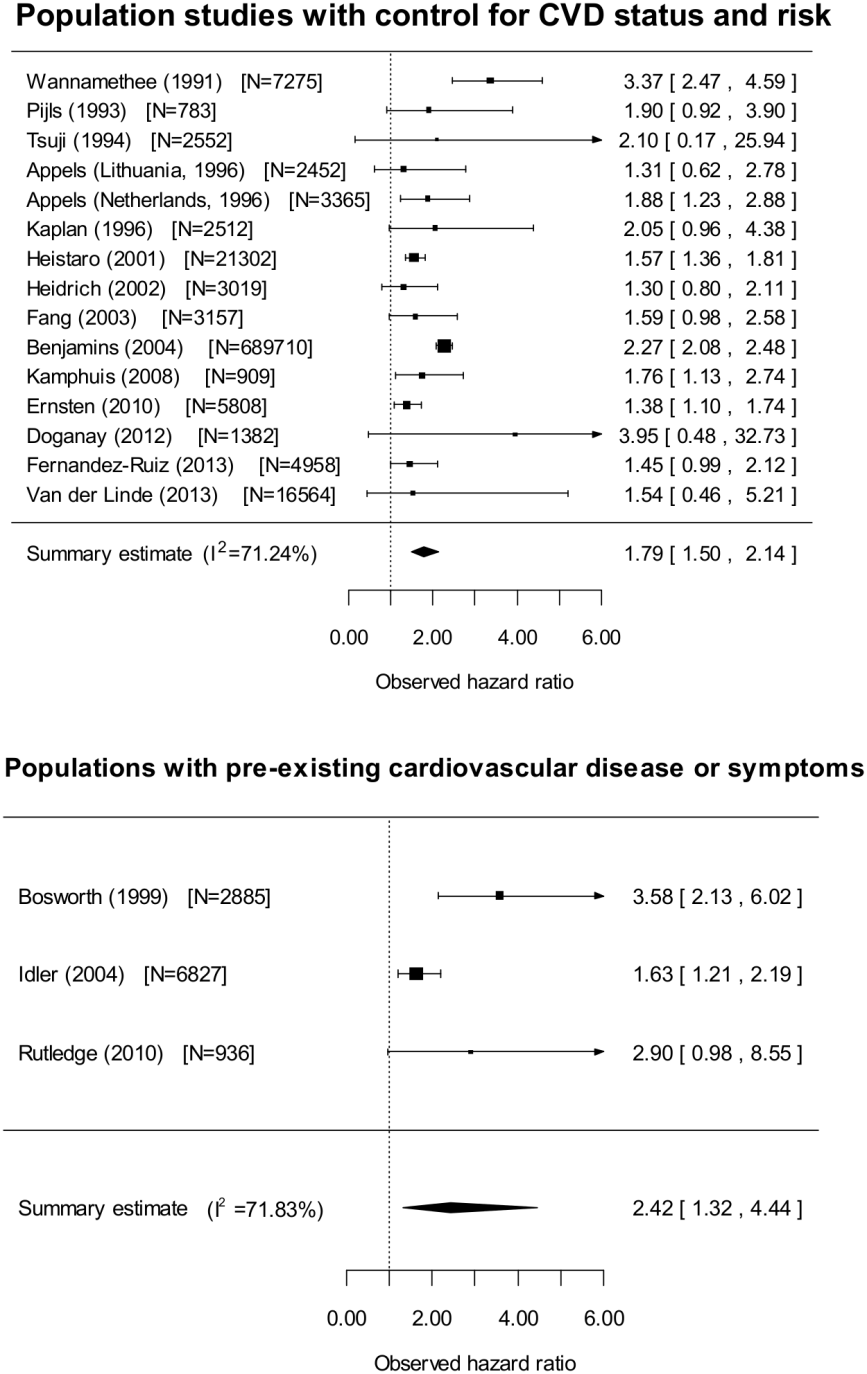
Meta-analysis of fatal CVD events in populations with varying degrees of control for CVD status and risk factors* and those with pre-existing disease: Poor health relative to excellent health.

Figure 3 shows the results of a meta-analysis of log-hazard ratios of poor health relative to excellent health for CVD mortality from studies of 15 unselected populations ranging from 783 to 689,710 participants where varying adjustments were made for baseline CVD status or risk. All studies were well-controlled for confounders except Wannamethee (1991) and Doğanay (2012) (see table 1). A weighted average was taken for the Wannamethee (1991) study because they reported results from different age subgroups and for the Heistaro (2001) and Ernsten (2011) studies, because they reported both male and female results. The combined summary estimate for the hazard ratio of poor relative to excellent health was 1.79 (95% CI 1.50 to 2.14), but there was significant heterogeneity as shown by the I^2^–statistic (I^2^ = 71.24%) and Q-test statistic (Q-test statistic of 48.7 (p<0.0001)). However, the I^2^ statistic was 0 if the meta-analysis was restricted to studies containing elderly populations, or to studies of men which were well controlled for covariates. In both cases, the summary estimate (hazard ratio) for CVD mortality for excellent to poor health was reduced. (See table 3). There was no significant effect of number of years of follow-up on the summary estimate (p = 0.059). The hazard ratio for CVD mortality was significantly lower as year of publication increased (p = 0.023), but still remained highly significant: HR 1.64 (95% CI 1.33 to 2.02, p<0.001) when including only studies published post-2000 in the meta-analysis.

Figure 3 also shows the results of a meta-analysis of log-hazard ratios of poor relative to excellent health for CVD mortality from studies of individuals with pre-existing cardiovascular disease or ischaemic heart disease symptoms. The combined summary estimate for the hazard ratio for these studies was 2.42 (95% CI 1.32 to 4.44), again with significant heterogeneity (Q test 7.10, p = 0.03). There was no significant effect of number of years of follow-up or year of publication on the summary estimate.

#### 2. Non-fatal CVD events

**Figure 4:**
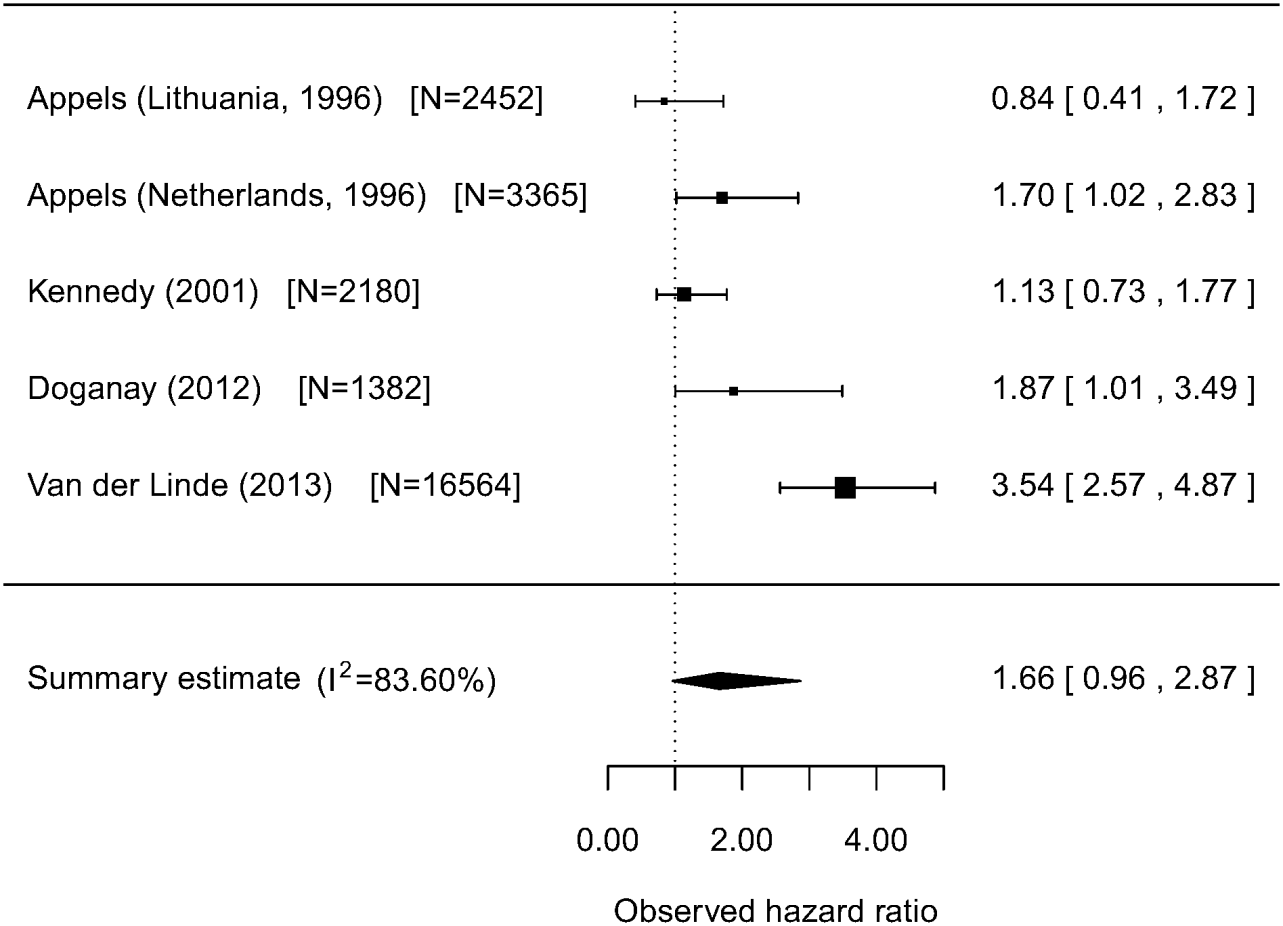
Meta-analysis of non-fatal CVD events in unselected populations with varying degrees of control for CVD status and risk factors: Poor health relative to excellent health.

Figure 4 shows the results of a meta-analysis of hazard ratios of poor health relative to excellent health for non-fatal CVD events from studies of unselected populations. The combined summary estimate for the hazard ratio was 1.66 (95% CI 0.96 to 2.87), and again there was significant heterogeneity, with a Q-test of 24.39, which was highly significant (p<0.0001). The heterogeneity, and hazard ratio was greatly reduced if the van der Linde study was omitted (table 3).

#### 3. Combined fatal and non-fatal CVD events

**Figure 5:**
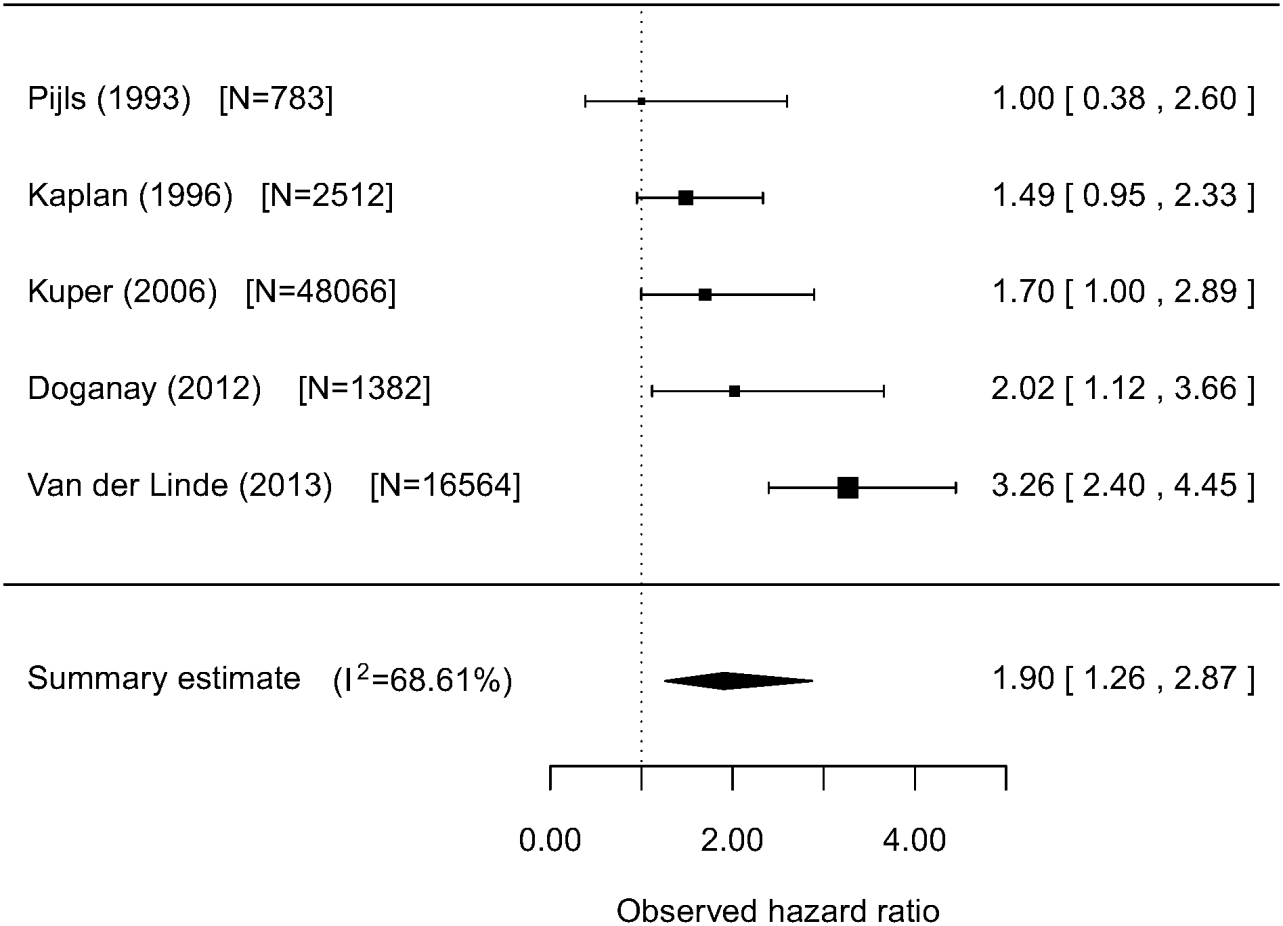
Meta-analysis of fatal and non-fatal CVD events in unselected populations with varying degrees of control for CVD status and risk factors: Poor health relative to excellent health.

Figure 5 shows the results of a meta-analysis of hazard ratios of poor health relative to excellent health for combined fatal and non-fatal CVD events from studies of unselected populations. The combined summary estimate for the hazard ratio was 1.90 (95% CI 1.26 to 2.87). The I^2^-statistic was 68.61%, falling to 0% without loss of significance after excluding van der Linde (2013) (see table 3). The Q-test for heterogeneity produced a test statistic of 12.74, which was significant (p = 0.013). There was no significant effect of number of years of follow-up on the summary estimate but the odds ratio for fatal and non-fatal CVD combined events was significantly higher for more recent publications (p = 0.012).

## Discussion

### Summary of findings

Our meta-analysis demonstrates a significant association between SRH and CVD mortality. This was present in populations with pre-existing disease and also in those where efforts were made to control for disease. Compared with excellent health, poor self-rated health was associated with more than one and a half times the risk of CVD mortality even after maximal adjustment for risk factors (including psychosocial factors, health behaviours and health service utilisation in some studies). This is comparable to but somewhat less than the effect size observed for SRH with all-cause mortality in other studies (HR for poor versus excellent 1.92 (95%CI (1.64, 2.25)).[1] The effect size (poor versus excellent SRH) was higher among those people with pre-existing cardiovascular disease or ischaemic heart disease symptoms; poor SRH was associated with more than twice the risk of CVD mortality after adjusting for disease severity in some studies. A significant relationship between SRH and cardiovascular outcome was also found in studies combining fatal and non-fatal CVD outcomes. While the observed association of SRH with non-fatal CVD events was of similar magnitude (HR 1.66), this was not statistically significant.

These findings need to be interpreted in the light of the observed heterogeneity. Both the size of the association with cardiovascular mortality and the degree of heterogeneity was reduced if the analysis was restricted to elderly populations, or male populations with good control of covariates. This suggests that our summary estimates of the association of SRH with cardiovascular mortality were probably inflated through inadequate adjustment for different participant characteristics in some of the studies. Heterogeneity in studies included in the meta-analysis of SRH and non-fatal CVD outcomes was considerably reduced by excluding van der Linde et al (2013), which found the strongest association between SRH and non-fatal CVD outcomes (3.54 95%CI (2.57,4.87)), but a weaker association with cardiovascular mortality than the other studies. The van der Linde study however was of high quality. It was carried out in a large population of over 16,000 men and women with over 10 years of follow-up, and with particular efforts made to exclude from the analyses those with pre-existing disease (i.e. reporting a diagnosis of stroke, heart attack or angina). Adjustments were also made for cardiovascular risk at baseline including behavioural factors and dietary measures, and outcomes were robustly defined and measured. Other studies had not excluded prevalent disease as thoroughly, or had included older cohorts where results were more likely to reflect current ill health. van der Linde also had more complete ascertainment of fatal CVD and of confirmed diagnosis of non-fatal CVD outcomes through hospital admission identified from participants’ National Health Service number through data linkage with Health Authority databases. This study therefore perhaps provides the most reliable estimates of the association of SRH with confirmed cardiovascular morbidity. Indeed, a more recent study not included in the meta-analysis due to statistical non-conformity with other included studies, also found a significant association between SRH and onset of non-fatal CVD (HR 1.25 for CHD and 1.54 for stroke p< = .001). In this study the strength of the association was closer to the other studies in this meta-analysis than to van der Linde, perhaps because it relied on a weaker method of ascertaining outcome (self report of physician diagnosis). [8].

### Limitations

There are still surprisingly few high quality studies in this area. We found only 5 cohorts reporting on the incidence or recurrence of non-fatal CVD, only 5 studies reporting combined fatal and non-fatal CVD events, and only 3 studies of fatal CVD events in those with pre-existing cardiovascular disease or ischaemic heart disease symptoms. There were methodological problems with many of the included studies, including poor measurement of baseline risk and CVD status (e.g. self-report versus objectively diagnosed), lack of detail of study methods and poor ascertainment of disease status and severity. We used a random-effects method of analysis because of the heterogeneity, but this results in smaller studies being given a greater relative weight than if a fixed effects method was performed.[35] This is a concern if the smaller studies were also those of the lowest quality. Nevertheless, we believe that our use of sensitivity analyses helped to address this problem. For a few studies where the 95% CI’s were not symmetric on the log-scale, the hazard ratios and 95% confidence intervals as presented in the forest plots do not exactly match those given in the original studies. This is because the hazard ratios and confidence intervals were transformed to the log-scale prior to analysis, with the process of extracting the standard error from the 95% CI on the log-scale and back-transformation resulting in slightly different numbers for the forest plots, mostly likely due to rounding or presentational error in the original studies. It was also not possible to adjust for later changes in covariates which may have taken place over the course of studies, and which may have impacted upon SRH (e.g. smoking and physical activity). There are also limitations with using the most extreme categories of SRH in each study in the meta-analysis. These include reducing the statistical power and potentially producing higher hazard ratios due to discarding a proportion of the sample. Finally, the clinical significance of the reported relationships is unknown, particularly in light of CVD being a heterogeneous category of conditions including coronary heart disease, and stroke with overlapping but distinct pathophysiological processes.

### SRH, mortality and disease

The association of SRH with CVD mortality found in our meta-analysis is likely to explain some but not all of the relationship between SRH and all-cause mortality. It nevertheless remains an important finding, in particular in view of the fact that in many of the studies included in the meta-analysis, adequate adjustment for covariates including traditional Framingham CV risk factors was undertaken. The most important hypothesis to explain the relationship between SRH and all-cause or disease-specific mortality is that subjective assessment of SRH is also a sensitive measure of objective health status. If this is so, precision of measurement of disease correlates, and better adjustment should decrease the strength of the association, which studies including better measurement of covariates may account for. Our meta-analysis did confirm that the strength of the association was reduced (but remained significant) if only well-controlled studies for cardiovascular risk were included. Secondly the association between SRH and mortality outcomes may reflect a personal predisposition to better or worse health, not related to objective health status at the time of questioning. The predisposition might be rooted in a range of health-protective factors, including biophysical factors; immune responses or neuro-endocrine homoeostasis, behavioural factors; diet, physical activity, smoking - usually adjusted for in studies, and psycho-social factors (positive affect, sense of coherence, or coping style). [36]–[41] Thirdly, the relationship between self-rated health and mortality may be mediated by functional limitations and decline or disability resulting from morbidity, supported by the strong relationship observed between self-rated and functional health. [2], [42] Rutledge et al in their sample of women with CVD symptoms found that SRH scores most closely overlapped with functional impairment status. [31] Even those without known CVD may be aware of early changes in functional ability, which are then reflected in overall self-ratings. [4] The stronger association of SRH with mortality in those with pre-existing diagnosis of cardiovascular disease supports the hypothesis that participant knowledge of disease status may affect SRH and its association with mortality. [7], [31].

### Conclusions

This study suggests that standard CVD risk factors and disease severity measures may underestimate risks of clinical cardiac events and that self-ratings may convey additional knowledge that may not be completely captured by epidemiological or available clinical measurement. The use of SRH as an additional risk factor in traditional cardiovascular risk prediction models or as part of a simple non-invasive risk score acceptable to patients in primary care therefore requires further investigation. It is uncertain when would be the best time to measure SRH for purposes of accurate outcome prediction. There is also uncertainty around whether SRH is in fact modifiable and if any improvements in SRH may result in improved cardiovascular outcomes. Nevertheless, those with poor self-rated health at cardiovascular risk in both non-clinical and clinical populations may warrant additional input from health services to identify and address reasons for their low subjective health.

## Supporting Information

Checklist S1 **PRISMA Checklist.**
(DOC)

File S1 **Sample search strategy.**
(DOCX)
