# Supplementary material for: Relationship of Self-Rated Health with Fatal and Non-Fatal Outcomes in Cardiovascular Disease: A Systematic Review and Meta-Analysis
Source: PLoS One. 2014 Jul 30;9(7):e103509. doi: 10.1371/journal.pone.0103509 (PMC4116199; doi:10.1371/journal.pone.0103509)
Supplement: File S1 — Sample search strategy. (DOCX) [file pone.0103509.s002.docx]

Appendix: Sample search strategy (Entrez PubMed)

## A. SELF-RATED HEALTH

## (Quality of Life\ OR Health Status\ OR Health Status Indicators\) AND (Self-assessed health [TW] OR Self-rated health [TW] OR Perceived health [TW] OR Subjective health [TW] OR Self-evaluated health [TW] OR Global health [TW] OR Self-reported health [TW])

B. Coronary heart disease

## Heart Diseases/ OR Coronary Disease/ OR Coronary Arteriosclerosis/ OR Coronary Stenosis/ OR Myocardial Ischemia/ OR Angioplasty, Transluminal, Percutaneous Coronary/ OR Coronary Thrombosis/ OR Angina Pectoris/ OR Angina, Unstable/ OR Myocardial Infarction/ OR Angina Pectoris/ OR Angina, Unstable/ OR Coronary Artery Bypass/

*OR*

Coronary disease [TW] OR Angina [TW] OR Ischaemic heart [TW] OR Myocard* infarct* [TW] OR Angioplasty [TW] OR Coronary bypass OR re-infarction[TW]

*OR*

CHD/ OR ACS/ OR AMI/

*A AND B*
